# Effect of Modulating DLPFC Activity on Antisocial and Prosocial Behavior: Evidence From a tDCS Study

**DOI:** 10.3389/fpsyg.2020.579792

**Published:** 2021-01-13

**Authors:** Wanjun Zheng, Yuzhen Li, Hang Ye, Jun Luo

**Affiliations:** ^1^Center for Economic Behavior and Decision-Making, Zhejiang University of Finance and Economics, Hangzhou, China; ^2^School of Economics, Zhejiang University of Finance and Economics, Hangzhou, China

**Keywords:** antisocial behavior, prosocial behavior, the dorsolateral prefrontal cortex, inequality, transcranial direct stimulation

## Abstract

Antisocial behavior and prosocial behavior in the condition of inequality have long been observed in daily life. Understanding the neurological mechanisms and brain regions associated with antisocial and prosocial behavior and the development of new interventions are important for reducing violence and inequality. Fortunately, neurocognitive research and brain imaging research have found a correlation between antisocial or prosocial behavior and the prefrontal cortex. Recent brain stimulation research adopting transcranial direct current stimulation or transcranial magnetic stimulation has shown a causal relationship between brain regions and behaviors, but the findings are mixed. In the present study, we aimed to study whether stimulation of the DLPFC can change participants’ antisocial and prosocial behavior in the condition of inequality. We integrated antisocial and prosocial behavior in a unified paradigm. Based on this paradigm, we discussed costly and cost-free antisocial and prosocial behavior. In addition, we also measured participants’ disadvantageous and advantageous inequality aversion. The current study revealed an asymmetric effect of bilateral stimulation over the DLPFC on costly antisocial behavior, while such an effect of antisocial behavior without cost and prosocial behavior with and without cost were not observed. Moreover, costly antisocial behavior exhibited by men increased after receiving right anodal/left cathodal stimulation and decreased after receiving right cathodal anodal/left anodal stimulation compared with the behavior observed under sham stimulation. However, subjects’ inequality aversion was not influenced by tDCS.

## Introduction

Antisocial behavior mainly involves actions intended to reduce other individuals’ endowment or access to resources, although they do not benefit the antisocial individual directly and may even cost that individual his or her own endowment (Abbink and Sadrieh, 2009). To observe antisocial behavior, Zizzo and Oswald (2001) proposed an experimental research method to evaluate antisocial behavior, and a series of subsequent studies proved the universality of antisocial behavior (Abbink and Herrmann, 2011; Zizzo and Fleming, 2011; Sadrieh and Schröder, 2012; Zhang and Ortmann, 2016). Although the joy of destruction is one reason for antisocial behavior (Abbink and Sadrieh, 2009), individuals’ preference for equality is one of the most important reasons for their antisocial behavior. Specifically, successful and rich people are the main targets of antisocial behavior (Zizzo, 2003; Dawes et al., 2007). Antisocial behavior is also related to norm violation. Behavioral economists and social psychologists proposed that social norms constrain antisocial behavior and facilitate cooperation (Knoch and Fehr, 2007; Baumeister, 2014; Buckholtz, 2015). Clinical scientists found that antisocial behavior results from a deficit in the capacity to inhibit responses to threats and rewards, which is similar to norm-violating behavior (Dolan, 2012; Patrick et al., 2012). In addition, antisocial behavior, such as aggressive behavior, is an important element of nonhuman primate social behavior (de Almeida et al., 2015). Although displays of aggression in male-male competition are common in all species of primates, antisocial behavior is important in the process of intergroup resource defense, predation, and reproduction (Bernstein and Gordon, 1974; Plavcan, 2012).

Contrary to antisocial behavior, prosocial behavior mainly involves voluntary actions intended to help or benefit another individual or group of individuals (Eisenberg and Mussen, 1989). In the past 30 years, economists have verified the widespread existence of prosocial behaviors such as altruism, fairness, trust, cooperation, and reciprocity through various behavioral experiments, including ultimatum games, dictator games, trust games, and public goods games (Güth et al., 1982; Isaac and walker, 1988; Forsythe et al., 1994; Berg et al., 1995). Interestingly, prosocial behavior is not unique in humans but also exists in nonhuman primates such as chimpanzees, monkeys, and apes (de Waal and Suchak, 2010; Yamamoto et al., 2012; Gilbert and Basran, 2019). Spontaneous assistance among nonhuman primates is abundant (de Waal, 2008; Silk and House, 2012; Mercier et al., 2017), ranging from bringing a mouthful of water to an incapacitated individual to slowing down travel injured companions (Boesch, 1992; de Waal, 1997). However, prosocial behavior seems to be relatively fragile compared to the more robust prosocial behavior in humans (Drayton and Santos, 2014). Specifically, no evidence to date has shown that nonhuman primates respond negatively to advantageous inequity (Drayton and Santos, 2014). Moreover, experimental studies also show that people’s prosocial behavior is easily affected by endowment (Cameron, 1999), regional culture (Henrich et al., 2001; Buchan et al., 2004), religion (Chen and Tang, 2009), social identity (Eckel and Grossman, 2005; Chen and Li, 2009) and other related factors. As individuals’ prosocial behavior is an important factor in resolving inequality, the impact of the endowment gap on prosocial behavior has also been found in experimental research (Piff et al., 2010; Romaniuc et al., 2019).

In accordance with behavioral studies, recent neuroimaging studies have suggested that the decision-making process of antisocial and prosocial behavior largely relies on the function of different brain regions. Neuroscientific research has found many brain regions associated with antisocial behavior, including various regions within the prefrontal cortex (such as the dorsolateral prefrontal cortex (DLPFC) and ventromedial prefrontal cortex), insular cortex, anterior cingulate cortex, amygdala, and striatal areas (Krämer et al., 2007, 2011; Sanfey, 2007; Veit et al., 2010; White et al., 2013; Hare et al., 2014; Kolling et al., 2016). Further evidence from head injury and lesion studies shows that individuals with damage to the frontal cortex exhibit more antisocial behavior (Anderson et al., 1999). A series of neuropsychological studies also report the association between frontal lobe dysfunction and increased antisocial and aggressive behavior (Foster et al., 1993; Deckel et al., 1996; Brower and Price, 2001; Yang and Raine, 2009). Within the prefrontal cortex, the DLPFC has been proven to be a region implicated in antisocial behavior. As one region of the affect-controlling paralimbic system (Sterzer et al., 2005), Rubia et al. (2009) found that the DLPFC was activated during executive functions. Dalwani et al. (2011) showed that boys with antisocial substance dependence (ASD) had significantly lower gray matter than controls in the left DLPFC. Fairchild et al. (2013) also revealed that antisocial and aggressive behaviors were negatively correlated with the right DLPFC. A series of meta-analyses were also performed to evaluate the association between antisocial behavior and DLPFC behavior (Morgan and Lilienfeld, 2000; Ogilvie et al., 2011; Alegria et al., 2016).

Similar to studies of antisocial behavior, neuroscientific research has also found a wide range of brain regions associated with prosocial behavior, including various regions within the prefrontal cortex, anterior insula, anterior cingulate cortex, and amygdala (Decety and Jackson, 2004; Moll et al., 2006; Bos et al., 2012; Aimone et al., 2014; Gabay et al., 2014; Feng et al., 2015). Further evidence from brain damage and lesion studies shows that bilateral dorsomedial prefrontal lesions increased altruistic punishment, whereas lesions of the right perisylvian region and temporo-insular cortex decreased antisocial behavior (Haushofer and Fehr, 2008; Moll et al., 2018). Within the prefrontal cortex, a series of studies have explored the association of the DLPFC and prosocial behavior (Sanfey et al., 2003; Spitzer et al., 2007). The right DLPFC has been consistently associated with altruistic punishment, valuation judgments, and fairness (Greene et al., 2004; Moll et al., 2005; Guo et al., 2013), whereas the left DLPFC has been found to be more related to executive function and impulse control (Ochsner et al., 2002; Figner et al., 2010; Barbey et al., 2014). Moreover, Glass et al. (2016) found the bilateral DLPFC to be associated with altruism.

However, all of these studies allow us to identify the associations between the DLPFC and antisocial or prosocial behavior, although the direct causal relationship remains unknown. Brain stimulation technologies such as transcranial direct current stimulation (tDCS) and transcranial magnetic stimulation (TMS) provide a way to influence the activity of target brain regions and establish causal relationships between the behavior and target brain region. Regarding antisocial behavior, Dambacher et al. (2015b) demonstrated that one kind of antisocial behavior, proactive aggression, was reduced in men after inducing right DLPFC activity. Choy et al. (2018) found that bilateral anodal stimulation of the DLPFC decreased individuals’ likelihood of committing aggressive behavior. Nevertheless, Hortensius et al. (2012) revealed that participants who received left frontal cortex stimulation exhibited more antisocial behavior, while Dambacher et al. (2015a) found no significant effect on the upregulation of the inferior frontal cortex. Regarding prosocial behavior, Ruff et al. (2013) found that increasing and suppressing neural excitability of the right lateral prefrontal cortex separately with tDCS resulted in significant changes in prosocial behavior, but stimulation had the opposite effect on prosocial behavior with and without strategic consideration. In contrast, the disruption of the right but not the left DLPFC with TMS reduces subjects’ ability to override self-interest motives (Knoch et al., 2006). Strang et al. (2015) revealed that reducing the activity of the right DLPFC by using TMS led to a significant decrease in prosocial behavior, but a significant effect was not found by reducing the activity of the left DLPFC.

Clearly, the findings of associations between the DLPFC and social behavior by using tDCS and TMS are mixed. In the current study, we hypothesized that the right DLPFC and left DLPFC play different roles in antisocial or prosocial behavior. Moreover, most of the studies above adopted the Taylor Aggression Paradigm to investigate antisocial behavior and used the ultimate game and dictator game to investigate prosocial behavior. Here, we integrate antisocial and prosocial behavior in a unified paradigm. To be more specific, to investigate antisocial and prosocial behavior in a single examination, participants are allowed to decrease or increase others’ endowment by sacrificing their own endowment. Moreover, we discussed antisocial and prosocial behavior in cost and no-cost situations. Finally, it should be noted that whether stimulation of the DLPFC can change antisocial and prosocial behavior under the condition of inequality are necessary to be examined.

## Materials and Methods

### Subjects

We recruited a total of 180 healthy students (108 females; mean age of 20.46 years, ranging from 18 to 27 years) of Zhejiang University of Finance and Economics. All participants met the following conditions: right-handed; unfamiliar with tDCS; and no history of clinical impairments, psychiatric illness, or neurological disorders. The participants were randomly assigned to experiment 1 (*n* = 90, 54 females) or experiment 2 (*n* = 90, 54 females). In experiment 1 and experiment 2, the participants were randomly assigned to sham stimulation (*n* = 30, 18 females), right anodal and left cathodal tDCS (*n* = 30, 18 females), or right cathodal and left anodal tDCS (*n* = 30, 18 females) groups. Participants received a fixed show-up fee of 10 CNY (approximately 1.5 US dollars) in addition to the money they gained during the prosocial or antisocial task and the equality aversion task. The entire experiment lasted approximately 50 min; on average, participants received a payment of approximately 57.1 CNY (approximately 8.59 US dollars) from the tasks, ranging from 14 to 157.5 CNY according to their performance and the computer program. In experiment 1 and experiment 2, because the costly decision is always before the cost-free decisions, there may be an order effect. To further test whether there was an order effect, we added four treatments of behavioral experiments. We recruited a total of 120 healthy students (60 females; mean age of 21.25 years, ranging from 18 to 25 years) of Zhejiang University of Finance and Economics to participate the added four treatments of behavioral experiments. Every experiment lasted approximately 30 min; on average, participants received a payment of approximately 32 CNY (approximately 4.82 US dollars). Participants gave written informed consent before entering the study, which was approved by the Zhejiang University of Finance and Economics Ethics Committee. No participants reported any adverse side effects involving scalp pain or headaches.

### Transcranial Direct Current Stimulation

Transcranial direct current stimulation applied a weak direct current to the scalp *via* two saline-soaked surface sponge electrodes (35 cm^2^). The current was constant and delivered by a battery-driven stimulator (multichannel, noninvasive wireless tDCS neurostimulator, Starlab, Barcelona, Spain), which was controlled by a Bluetooth system. Generally, cathodal stimulation restrains cortical excitability, whereas anodal stimulation enhances it (Nitsche and Paulus, 2000).

**Figure 1:**
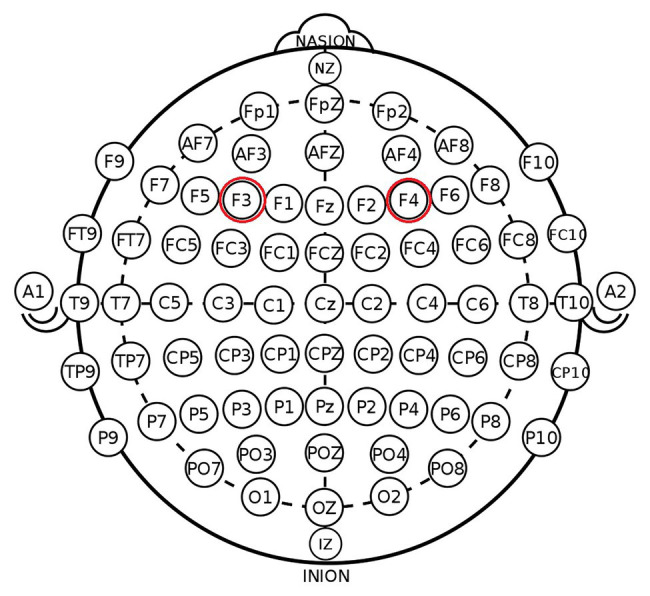
Schematic and locations of electrode positions.

**Figure 2:**
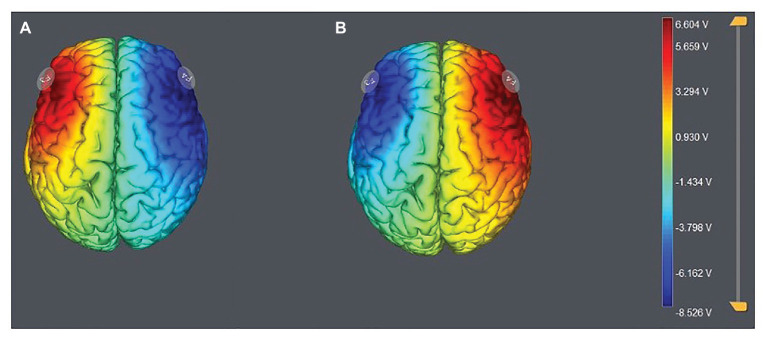
Stimulation modes of the two treatments. Anodal stimulation over the left DLPFC and cathodal stimulation over the right DLFPC **(A)** and anodal stimulation over the right DLPFC and cathodal stimulation over the left DLFPC **(B)**.

According to the EEG 10–20 system, we chose the right F4 and left F3 positions to place the electrodes (Figure 1). The participants were randomly assigned to one of the three stimulation treatments: anodal stimulation over the right DLPFC and cathodal stimulation over the left DLPFC, anodal stimulation over the left DLPFC and cathodal stimulation over the right DLPFC (Figure 2), and sham stimulation. A constant current of 3 mA (1.5 mA to each DLPFC site) was applied for 20 min. Following the standard tDCS protocol, stimulation commenced after a 30-s ramp-up period, and the current was ramped down over the last 30 s. For sham stimulation, the current lasted only 30 s. This approach has proven to be reliable because the brief duration of stimulation could hardly modulate cortical excitability, but the participants may feel the initial itching and believe they were receiving stimulation (Gandiga et al., 2006).

### Experimental Task and Procedure

#### Experiment 1 and Experiment 2

In experiment 1, we designed an antisocial task with and without cost. The antisocial task contained two stages. In the first stage, participants were given 10 or 30 tokens randomly. The participant who was given 10 tokens was the decision-maker, and the participant who was given 30 tokens was the recipient. The decision-maker could decrease the recipient’s endowment by giving up their own endowment at a rate of 1:3. In the second stage, participants were also given 10 or 30 tokens randomly. The participant who was given 10 tokens was the decision-maker, and the participant who was given 30 tokens was the recipient. The decision-maker could decrease the recipient’s endowment without cost. The recipient’s endowment could be reduced to zero but could not become negative. Overall, the difference between the two stages was that decision-makers could decrease recipients’ endowment at a personal cost or without cost.

In experiment 2, we designed a prosocial task with and without cost. The prosocial task also contained two stages. In the first stage, participants were given 30 or 10 tokens randomly. The participant who was given 30 tokens was the decision-maker, and the participant who was given 10 tokens was the recipient. The decision-maker could increase the recipient’s endowment by giving up their own endowment at a rate of 1:3. In the second stage, participants were also given 10 or 30 tokens randomly. The participant who was given 30 tokens was the decision-maker, and the participant who was given 10 tokens was the recipient. The decision-maker could increase the recipient’s endowment without cost. The recipient’s endowment could be increased by 30 at most. In sum, the difference between the two stages was that decision-makers could increase recipients’ endowment at a personal cost or without cost.

In particular, at the beginning of stage 1 or stage 2, participants did not know their roles. Each participant made a decision as if he or she was the decision-maker. Each participant could increase or decrease the recipient’s endowment in the prosocial or antisocial experiment as if he or she was the decision-maker. Then, each participant’s role was randomly determined by the computer. If a participant’s role was a decision-maker, her partner’s endowment increased or decreased according to her decision. If a participant’s role was a recipient, her endowment increased or decreased according to her partner’s decision.

### Inequality Aversion Task

The prosocial or antisocial task was followed by an inequality aversion task. We used the measurement method proposed by Yang et al. (2016) to obtain the participants’ advantageous inequality aversion and disadvantageous inequality aversion data. We adopted menu 1 (Yang et al., 2016), which consisted of 10 choices, to measure the degree of the participants’ disadvantageous inequality aversion. Each choice had two different options (A and B), and each option distributed money to the decision-maker and to another anonymous participant. In option A of the 10 choices, the decision-maker’s endowment dropped from 125 tokens to 35 tokens, and the recipient’s endowment of 150 tokens remained unchanged. In option B of the 10 choices, the decision-maker’s endowment of 100 tokens remained unchanged, and the recipient’s endowment of 260 remained unchanged. The decision-maker was the same participant for each of the 10 choices. In each of the payoff pairs in menu 1, the decision-maker’s payoff was lower than the recipient’s payoff. Therefore, the more option A was chosen, the greater the participant’s degree of disadvantageous inequality aversion.

In addition, we adopted menu 2 (Yang et al., 2016), which also consisted of 10 choices, to measure the degree of the participants’ advantageous inequality aversion. Similar to menu 1, each choice had two different options (A and B), and each option distributed money to the decision-maker and to another anonymous participant. In contrast to menu 1, the payoff of the decision-maker was higher than that of the recipient for all choices. In option A of the 10 choices, the decision-maker’s endowment dropped from 185 tokens to 35 tokens, and the recipient’s endowment of 90 tokens remained unchanged. In option B of the 10 choices, the decision-maker’s endowment of 170 tokens remained unchanged, and the recipient’s endowment of 50 tokens remained unchanged. Therefore, the more option A was chosen, the greater the participant’s degree of advantageous inequality aversion.

### Experimental Procedure

**Figure 3:**
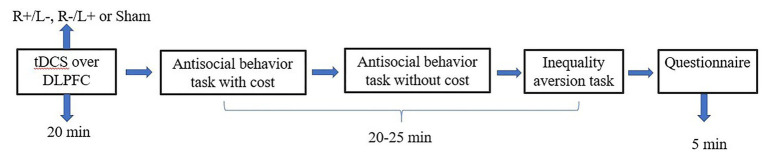
Schematic representation of the experimental design in experiment 1. After 20 min of stimulation, the participant was asked to complete the costly antisocial behavior task, the antisocial behavior without cost task, and the inequality aversion task.

**Figure 4:**
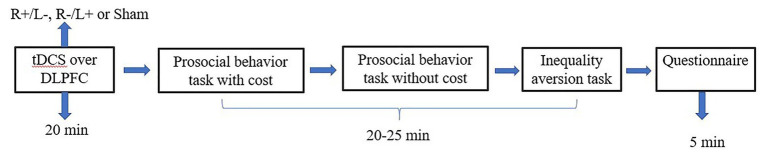
Schematic representation of the experimental design in experiment 2. After 20 min of stimulation, the participant was asked to complete the costly prosocial behavior task, the prosocial behavior without cost task, and the inequality aversion task.

We used the experimental software z-Tree to present the tasks as well as to automatically calculate the final payoff. The entire experiment was conducted in three stages (Figures 3, 4). In the first stage, participants received one of three stimulation patterns for 20 min. In the second stage, participants had to pass a control question test to ensure that they fully understood the profit outcomes of their decisions. Then, participation in antisocial treatment decided the amount they wanted to decrease recipient tokens with or without cost, and participation in prosocial treatment decided the amount they wanted to increase recipient tokens with or without cost. After that, participants completed the inequality aversion measurement. In the third stage, participants were asked to complete a questionnaire before they finally received their payment. The questionnaire contained questions about personal information, such as sex, age, income, and consumption expenditure. The participants were informed about how their decisions determined their final payments; the game was played once with each participant randomly paired with another participant, and in the second stage of the experiment, the role each participant played in this game was also randomly assigned by the computer.

To further test whether there was an order effect, we added four treatments of behavioral experiments. In the first two treatments of experiments, participants first completed a prosocial or antisocial task with cost and then completed a prosocial (*n* = 30, 15 females) or antisocial (*n* = 30, 15 females) task without cost. In the latter two treatments of experiments, participants first completed the prosocial or antisocial task without cost and then completed the prosocial (*n* = 30, 15 females) or antisocial (*n* = 30, 15 females) task with cost.

### Data Analysis

To assess the effects of anodal and cathodal brain stimulation on antisocial or prosocial behavior with and without cost, we ran regression analyses in STATA software. These analyses predicted for each individual *i* the observed decision (shifts in token count) with the following equation.

(1) $$y=\beta_{0}+\beta_{1}*D_{i}+\beta_{2}*X_{j}+\varepsilon_{i}$$

For the analysis of antisocial and prosocial behavior with cost, y is given by the shifts in token count in stage 1 in antisocial or prosocial experiment. For the analysis of antisocial and prosocial behavior without cost, y is given by the number of tokens the decision-maker chooses to increase the recipient’s endowment in stage 2 in antisocial or prosocial experiment. D_i_ are dummy-coded variables that are set to 1 if individual i received stimulation of right anodal/left cathodal, right cathodal/left anodal, or sham, respectively. Thus, the parameters $\beta_{1}$ quantify the change in antisocial or prosocial behavior with or without cost due to right anodal/left cathodal and right cathodal/left anodal, relative to the (omitted) sham group. In addition, the parameters $\beta_{1}$ also quantify the change in behavior due to right anodal/left cathodal or sham tDCS relative to the (omitted) right cathodal/left anodal group. Participants were asked to complete a questionnaire that contained questions about personal information such as sex, age, income, consumption expenditure, and the educational level of parents. The model further contained $\beta_{j}$ to capture the effects of personal characteristics under the above three stimulation conditions.

In addition to regression analysis, we also conducted Kruskal–Wallis test to determine whether there were differences in the number of tokens offered among the three kinds of stimulations. When a significant difference was found, the Mann–Whitney U test was run to compare the concrete difference.

All data were statistically evaluated using Stata software. The significance level was set at 0.05 for all analyses. The means and standard errors of the prosocial behaviors and antisocial behaviors at a cost of 1:3 tokens are shown in Table 1. Additionally, the means and standard errors of the prosocial behaviors and antisocial behaviors without cost are shown in Table 1.

**Table 1:** Means and SE of the data for prosocial and antisocial behavior with and without cost.

Cost	Behavior	R anodal/L cathodal	L anodal/R cathodal	Sham	Total
With cost	Prosocial	3.61 (0.61)	3.03 (0.41)	2.95 (0.48)	3.19 (0.28)
	Antisocial	2.05 (0.47)	0.72 (0.27)	1.43 (0.35)	1.40 (0.22)
Without cost	Prosocial	25.77 (1.40)	25.53 (1.03)	25.83 (1.22)	25.69 (0.69)
	Antisocial	13.17 (1.90)	14.82 (2.08)	12.93 (1.71)	13.64 (1.09)

## Results

### Antisocial Task

#### tDCS Effect

##### Antisocial Behavior at a Cost of 1:3 Tokens

We first regressed antisocial behavior at a cost of 1:3 using group as an independent variable. The regression results are shown in columns 1 and 2 of Table 2. We found that R anodal/L cathodal stimulation significantly increased antisocial behavior compared with R cathodal/L anodal stimulation (*p* = 0.013). However, the change in antisocial behavior with cost due to R+/L− or R−/L+ tDCS relative to the (omitted) sham group was not significant. Furthermore, we contain the effects of personal characteristics in the regression model and the results are shown in columns 3 and 4 of Table 2. The results indicated that the impact of tDCS was robust.

**Table 2:** Antisocial behavior with cost.

Regressor	Base group: sham Coeff. (*p*)	Base group: R−/L+ Coeff. (*p*)	Base group: sham Coeff. (*p*)	Base group: R−/L+ Coeff. (*p*)
R+/L−	0.616 (0.246)	1.333 (0.013)	0.578 (0.287)	1.248 (0.025)
R−/L+	−0.717 (0.178)	---	−0.669 (0.220)	---
Sham	---	−0.717 (0.178)	---	0.669 (0.220)
Sex	---	---	−0.549 (0.221)	−0.549 (0.221)
Age	---	---	0.132 (0.480)	0.132 (0.480)
Mother education	---	---	−0.355 (0.265)	−0.355 (0.265)
Father education	---	---	0.5723 (0.082)	0.5723 (0.082)
Family income	---	---	−0.154 (0.408)	−0.154 (0.408)
Consumption	---	---	0.147 (0.593)	0.147 (0.593)
Constant	1.433 (0.0001)	0.717 (0.058)	−1.597 (0.698)	−2.266 (0.582)

**Figure 5:**
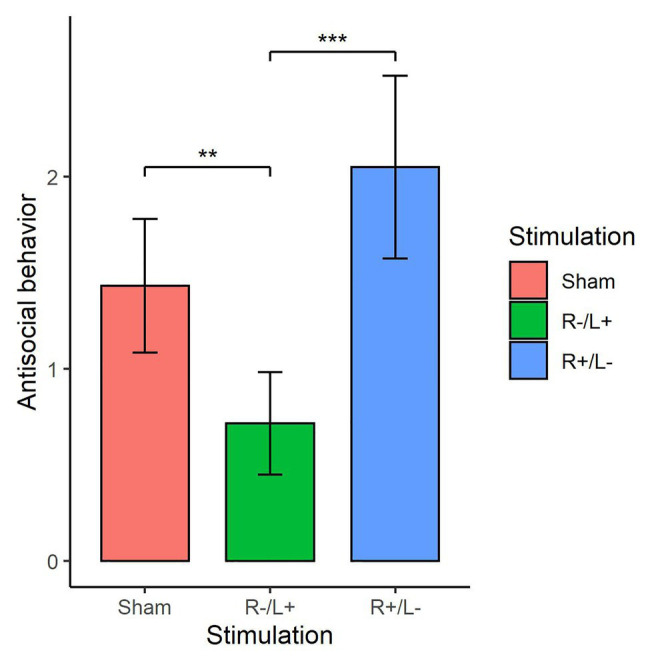
Antisocial behavior at a cost of 1:3 tokens in three stimulation conditions. Error bars indicate 95% CI. Asterisks indicate significant differences in behavior between treatments.

We further utilized the Kruskal–Wallis test to verify whether there were differences in the number of tokens offered among the three stimulation conditions. Figure 5 shows antisocial behavior under different stimulation conditions. The Kruskal–Wallis test showed that there was a significant difference in antisocial behavior under the three different stimulation conditions ($\chi_{d.f.2}^{2}=7.911$, *p* = 0.019). The Mann–Whitney U test showed that the antisocial behavior in R cathodal/L anodal stimulation significantly decreased compared with the antisocial behavior observed under sham stimulation (*z* = 1.973, *p* = 0.048). The Mann–Whitney U test also showed that the antisocial behavior under R cathodal/L anodal stimulation significantly decreased compared with the antisocial behavior observed under R anodal/L cathodal stimulation (*z* = 2.759, *p* < 0.01).

##### Antisocial Behavior Without Cost

We further regressed antisocial behavior without using the group as an independent variable. The regression results are shown in columns 1 and 2 of Table 3. We found that the effect of tDCS on antisocial behavior was not significant. The results were robust after containing the effects of personal characteristics in the regression model (the results are shown in columns 3 and 4 of Table 3).

**Table 3:** Antisocial behavior without cost.

Regressor	Base group: sham Coeff. (*p*)	Base group: R−/L+ Coeff. (*p*)	Base group: sham Coeff. (*p*)	Base group: R−/L+ Coeff. (*p*)
R+/L−	0.233 (0.931)	−1.653 (0.541)	−0.775 (0.777)	−2.892 (0.298)
R−/L+	1.887 (0.486)	------	2.116 (0.442)	------
Sham	------	−1.887 (0.486)	------	−2.116 (0.442)
Sex	------	------	−0.403 (0.076)	−0.403 (0.076)
Age	------	------	1.702 (0.073)	1.702 (0.073)
Mother education	------	------	−2.021 (0.209)	−2.021 (0.209)
Father education	------	------	1.954 (0.238)	1.954 (0.238)
Family income	------	------	0.852 (0.368)	0.852 (0.368)
Consumption	------	------	0.077 (0.955)	0.077 (0.955)
Constant	12.933 (0.0001)	14.82 (0.0001)	−23.171 (0.267)	−21.054 (0.312)

We also utilized the Kruskal–Wallis test to verify whether there were differences in the number of tokens offered among the three stimulation conditions. The Kruskal–Wallis test showed that there was no significant difference in antisocial behavior under the three different stimulation conditions ($\chi_{d.f.2}^{2}=0.753$, *p* = 0.683).

### Prosocial Task

#### tDCS Effect

##### Prosocial Behavior at a Cost of 1:3 Tokens

We further regressed prosocial behavior at a cost of 1:3 using group as an independent variable. The regression results are shown in columns 1 and 2 of Table 4. We found that the effect of tDCS on prosocial behavior with cost was not significant. The results were robust after containing the effects of personal characteristics in regression model (the results are shown in columns 3 and 4 of Table 4).

**Table 4:** Prosocial behavior with cost.

Regressor	Base group: sham Coeff. (*p*)	Base group: R−/L+ Coeff. (*p*)	Base group: sham Coeff. (*p*)	Base group: R−/L+ Coeff. (*p*)
R+/L−	0.651 (0.576)	0.307 (0.786)	0.872 (0.478)	0.124 (0.918)
R−/L+	0.344 (0.757)	−0.344 (0.757)	0.749 (0.526)	−0.749 (0.526)
Sham	---	---	---	---
Sex	---	---	1.219 (0.207)	1.219 (0.207)
Age	---	---	0.057 (0.790)	0.057 (0.790)
Mother education	---	---	−0.295 (0.606)	−0.295 (0.606)
Father education	---	---	−0.083 (0.892)	−0.083 (0.892)
Family income	---	---	0.048 (0.917)	0.048 (0.917)
Consumption	---	---	0.689 (0.239)	0.689 (0.239)
Constant	3.592 (0.0001)	3.936 (0.0001)	0.446 (0.923)	1.195 (0.797)

We also conducted the Kruskal–Wallis test to verify whether there were differences in the amount offered among the three stimulation conditions. The Kruskal–Wallis test showed that there was no significant difference in prosocial behavior under the three different stimulation conditions ($\chi_{d.f.2}^{2}=0.024$, *p* = 0.887).

##### Prosocial Behavior Without Cost

We further regressed prosocial behavior without cost using group as an independent variable. The regression results are shown in columns 1 and 2 of Table 5. We find that the effect of tDCS on prosocial behavior without cost was not significant. The results were robust after containing the effects of personal characteristics in regression model (the results are shown in columns 3 and 4 of Table 5).

**Table 5:** Prosocial behavior without cost.

Regressor	Base group: sham Coeff. (*p*)	Base group: R−/L+ Coeff. (*p*)	Base group: sham Coeff. (*p*)	Base group: R−/L+ Coeff. (*p*)
R+/L−	−0.053 (0.976)	0.247 (0.882)	−1.208 (0.497)	−0.905 (0.597)
R−/L+	−0.301 (0.860)	---	−0.303 (0.862)	---
Sham	---	−0.301 (0.860)	---	0.303 (0.862)
Sex	---	---	2.739 (0.061)	2.739 (0.061)
Age	---	---	0.852 (0.065)	0.852 (0.065)
Mother education	---	---	−0.635 (0.452)	−0.635 (0.452)
Father education	---	---	1.310 (0.139)	1.310 (0.139)
Family income	---	---	0.748 (0.246)	0.748 (0.246)
Consumption	---	---	−1.650 (0.048)	−1.650 (0.048)
Constant	25.827 (0.0001)	25.526 (0.0001)	8.050 (0.399)	7.747 (0.420)

We also conducted the Kruskal–Wallis test to verify whether there were differences in the number of tokens offered between the three stimulation conditions. The Kruskal–Wallis test showed that there was no significant difference in prosocial behavior without cost under the three different stimulation conditions ($\chi_{d.f.2}^{2}=0.753$, *p* = 0.683).

### Sex

We further tested the effect of sex on antisocial and prosocial behavior under the three different stimulation conditions.

**Figure 6:**
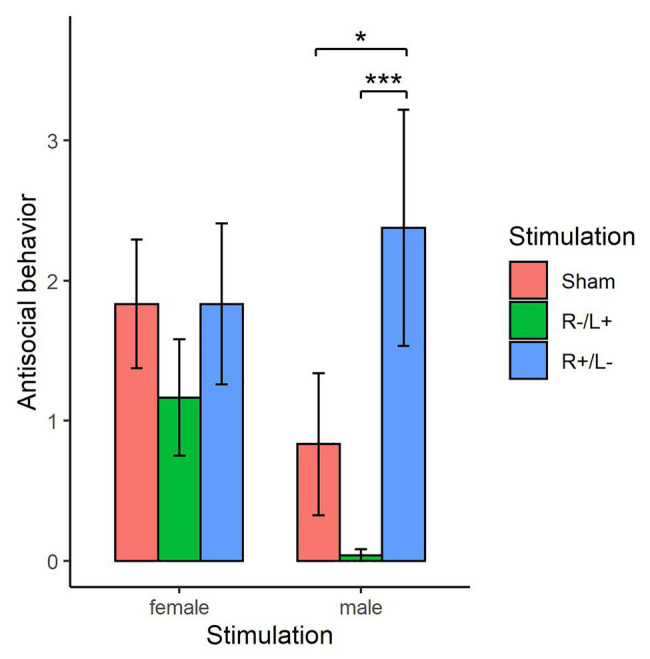
Antisocial behavior exhibited by females and males at a cost of 1:3 tokens under three stimulation conditions. Error bars indicate 95% CI. Asterisks indicate significant differences in behavior among treatments.

The Shapiro–Wilk test showed that antisocial behavior exhibited by females or males at a cost of 1:3 tokens was not normally distributed (*p* < 0.01). Figure 6 shows the antisocial behavior of females and males at a cost of 1:3 tokens under different stimulation conditions separately. For females, the Kruskal–Wallis test showed that there was no significant difference in antisocial behavior under the three different stimulation conditions ($\chi_{d.f.2}^{2}=2.163$, *p* = 0.339). For males, the Kruskal–Wallis test showed that there was a significant difference in antisocial behavior under the three different stimulation conditions ($\chi_{d.f.2}^{2}=9.945$, *p* < 0.01). The Mann–Whitney U test showed that antisocial behavior under R anodal/L cathodal stimulation significantly increased compared with that observed under sham stimulation (*z* = −1.796, *p* = 0.073). The Mann–Whitney U test also showed that antisocial behavior under R cathodal/L anodal stimulation significantly decreased compared with that observed under R anodal/L cathodal stimulation (*z* = −3.055, *p* < 0.01). However, antisocial behavior under R cathodal/L anodal stimulation was not significantly changed compared with that observed under sham stimulation (*z* = 1.200, *p* = 0.230).

The Shapiro–Wilk test showed antisocial behavior without cost exhibited by females or males was not normally distributed (*p* < 0.01). For females or males, the Kruskal–Wallis test showed that there was no significant difference in antisocial behavior under the three different stimulation conditions ($\chi_{d.f.2}^{2}=2.631$, *p* = 0.268;$\chi_{d.f.2}^{2}=0.334$, *p* = 0.846).

The Shapiro–Wilk test showed that prosocial behavior at a cost of 1:3 exhibited by females or males was not normally distributed (*p* < 0.01). For females or males, the Kruskal–Wallis test showed that there was no significant difference in prosocial behavior under the three different stimulation conditions ($\chi_{d.f.2}^{2}=1.592$, *p* = 0.451;$\chi_{d.f.2}^{2}=4.308$, *p* = 0.116). The Shapiro–Wilk test showed that prosocial behavior without cost exhibited by females or males was not normally distributed (*p* < 0.01). For females or males, the Kruskal–Wallis test showed that there was no significant difference in antisocial behavior under the three different stimulation conditions ($\chi_{d.f.2}^{2}=0.080$, *p* = 0.961; $\chi_{d.f.2}^{2}=0.279$, *p* = 0.867).

#### Inequality Aversion Task

The Shapiro–Wilk test showed that disadvantageous inequality and advantageous inequality in antisocial behavior and prosocial behavior were not normally distributed (*p* < 0.01). In antisocial behavior, the Kruskal–Wallis test showed that there was no significant difference in disadvantageous inequality and advantageous equality under the three different stimulation conditions ($\chi_{d.f.2}^{2}=2.093$, *p* = 0.351; $\chi_{d.f.2}^{2}=2.167$, *p* = 0.338). In prosocial behavior, the Kruskal–Wallis test also showed that there was no significant difference in disadvantageous inequality and advantageous equality ($\chi_{d.f.2}^{2}=0.029$, *p* = 0.986; $\chi_{d.f.2}^{2}=0.092$, *p* = 0.955).

#### Correlations Between Behavior and Inequality Aversion

Spearman’s test was conducted to elucidate the correlation between behavior and inequality aversion. Spearman’s test results showed that antisocial behavior at a cost of 1:3 tokens was positively correlated with disadvantageous inequality aversion (Spearman’s rho = 0.413, *p* < 0.01) but not with advantageous inequality aversion (Spearman’s rho = −0.002, *p* = 0.98). Antisocial behavior without cost was positively correlated with disadvantageous inequality aversion (Spearman’s rho = 0.408, *p* < 0.01) and negatively correlated with advantageous inequality aversion (Spearman’s rho = −0.177, *p* = 0.095). Furthermore, Spearman’s test results showed that prosocial behavior at a cost of 1:3 tokens was positively correlated with advantageous inequality aversion (Spearman’s rho = 0.353, *p* < 0.01) and was negatively correlated with disadvantageous inequality aversion (Spearman’s rho = −0.172, *p* = 0.090). Prosocial behavior without cost was positively correlated with advantageous inequality aversion (Spearman’s rho = 0.382, *p* < 0.01) and negatively with advantageous inequality aversion (Spearman’s rho = −0.337, *p* < 0.01).

#### Order Effect

The Mann–Whitney U test indicated that antisocial behavior without cost was not influenced by the order (*z* = −0.717, *p* = 0.473). Similarly, the Mann–Whitney U test also revealed that prosocial behavior without cost was not influenced by the order (*z* = −0.618, *p* = 0.537). In addition, the Mann–Whitney U test showed that antisocial and prosocial behavior with cost were also not influenced by the order (*z* = −0.185, *p* = 0.853; *z* = −0.523, *p* = 0.601).

## Discussion

A series of previous studies from different fields have discussed the issues of antisocial behavior and prosocial behavior. Many brain regions, such as the prefrontal cortex, insular cortex, anterior cortex, amygdala, and striatal areas (Decety and Jackson, 2004; Moll et al., 2006; Krämer et al., 2007, 2011; Veit et al., 2010; Bos et al., 2012; White et al., 2013; Aimone et al., 2014; Gabay et al., 2014; Hare et al., 2014; Feng et al., 2015; Kolling et al., 2016), are implicated in the two types of social behavior. Moreover, a great deal of neuroscientific and head injury studies and documents have revealed that the DLPFC is an important region correlated with antisocial and prosocial behavior (Morgan and Lilienfeld, 2000; Sanfey et al., 2003; Greene et al., 2004; Moll et al., 2005; Spitzer et al., 2007; Dalwani et al., 2011; Ogilvie et al., 2011; Fairchild et al., 2013; Guo et al., 2013; Alegria et al., 2016; Glass et al., 2016). To find the association between these forms of social behavior and brain regions, researchers often used tDCS and TMS to change the activity of the cortex (Ruff et al., 2013; Dambacher et al., 2015a; Strang et al., 2015). However, the previous findings are mixed (Choy et al., 2018), and it is worth noting that antisocial and prosocial behavior under the condition of inequality are seldom examined.

The present research complements these studies with tDCS by providing a causal relationship between antisocial or prosocial behavior in the condition of inequality and the activities of the DLPFC. The findings of the present research contribute to reinforcing conclusions from neuroimaging and brain stimulations by experimentally documenting the role of the DLPFC on the possibility of engaging antisocial or prosocial behavior and the perception of inequality aversion (Morgan and Lilienfeld, 2000; Ogilvie et al., 2011; Fairchild et al., 2013; Alegria et al., 2016). Our findings also show the feasibility of combining brain stimulation with social psychological manipulation to reduce antisocial behavior.

We observed an asymmetric effect of bilateral stimulation over the DLPFC on the subjects’ costly antisocial behavior. According to the data in the treatment of antisocial behavior at a cost of 1:3 tokens, subjects’ costly antisocial behavior decreased when the activity of the right DLPFC was restrained and the activity of the left DLPFC was improved. In contrast, subjects’ costly antisocial behavior increased when they received right anodal/left cathodal tDCS over the DLPFC compared with right cathodal anodal/left anodal stimulation. This seems to indicate that the left and right DLPFC play different roles in costly antisocial behavior, which is consistent with the findings of previous studies (Hortensius et al., 2012; Dambacher et al., 2015b).

However, we did not observe such an asymmetric effect of bilateral stimulation over the DLPFC on the subjects’ antisocial behavior without cost. According to the data in the treatment of antisocial behavior without cost, we did not observe any significant change in subjects’ antisocial behavior under the three different stimulations, which is inconsistent with the findings of the study of the association between aggressive behavior and inferior frontal cortex (Dambacher et al., 2015a). Together, the results of antisocial behavior described above seem to indicate that the DLPFC has different roles in antisocial behavior depending on the cost.

Previous studies have shown that costly behavior is different from cost-free behavior. McAuliffe et al. (2015) revealed that children’s third-party punishment behavior decreased when punishment behavior was personally costly. Our data also supported that the level of costly antisocial and prosocial behavior was lower than the level of cost-free behavior. Moreover, according to our data regarding prosocial behavior at a cost of 1:3 tokens, we found that subjects’ costly prosocial behavior increased slightly when receiving right anodal/left cathodal tDCS over the DLPFC compared with that observed when receiving right cathodal anodal/left anodal stimulation or sham stimulation. However, this increase was not significant. This finding is partly consistent with a previous study that revealed that there was no significant effect of TMS on participants’ prosocial behavior by stimulating the left DLPFC (Strang et al., 2015). In addition, evidence from the study by Strang also showed that the effect of stimulating the left DLPFC on prosocial behavior was not significant when strategic consideration occurred (Strang et al., 2015). Similarly, according to the data for prosocial behavior without cost, we did not observe any significant change in subjects’ antisocial behavior under the three different conditions. Together with the results of antisocial behavior, these results seem to indicate that the DLPFC plays different roles in antisocial and prosocial behavior.

In the experimental procedure, because the costly decisions are always before the cost-free decisions, there may be an order effect. It is certain that the costly decision is not influenced because participants did not know any details of the costly free decisions before they completed the antisocial or prosocial task at a cost. However, cost-free decisions may be influenced by the order effect. To reduce the order effect, we arranged that participants did not know the results of the antisocial or prosocial tasks at a cost when they made cost-free decisions. To be more specific, all the tasks’ profits were shown to participants at the end of the experiment. In addition, participants were repaired in pairs in the antisocial or prosocial task without cost. Thus, every participant’s partner was different between costly decisions and cost-free decisions. Certainly, that is not enough to completely avoid the order effect. To further test whether there was an order effect, we added four treatments of behavioral experiments. The Mann–Whitney U test indicated that antisocial behavior without cost was not influenced by the order (*z* = −0.717, *p* = 0.473). Similarly, the Mann–Whitney U test also revealed that prosocial behavior without cost was not influenced by the order (*z* = −0.618, *p* = 0.537). In addition, the Mann–Whitney U test showed that antisocial and prosocial behavior with cost were also not influenced by the order (*z* = −0.185, *p* = 0.853; *z* = −0.523, *p* = 0.601). However, further research should pay attention to counterbalancing antisocial and prosocial tasks with and without cost.

We also explored sex differences with respect to the tDCS effect. The results suggested that the cortical excitability of the DLPFC has a significant effect on males regarding costly antisocial behavior, but the effect was not significant on females. The costly antisocial behavior exhibited by men receiving right anodal/left cathodal stimulation increased compared with the costly antisocial behavior observed under sham stimulation. In addition, the costly antisocial behavior exhibited by men receiving right cathodal anodal/left anodal stimulation decreased compared with that observed under sham stimulation. Previous literature has suggested that men display more aggression tendencies and physically aggressive behavior than women (Björkqvist, 1994; Archer, 2004; Dambacher et al., 2015b). Our findings indicated that antisocial behavior could be influenced by stimulation. However, this kind of effect was not significant for antisocial behavior without cost, costly prosocial behavior, or prosocial behavior without cost.

To further interpret the mechanism of antisocial and prosocial behavior, attention was paid to inequality aversion. Spearman’s test indicated that costly antisocial behavior was positively correlated with disadvantageous inequality aversion and antisocial behavior without cost was positively correlated with disadvantageous inequality. This is inconsistent with the findings that inequality aversion predicts antisocial punishment in public goods games with punishment (Thöni, 2014). Additionally, costly prosocial behavior was positively correlated with advantageous inequality aversion. Prosocial behavior without cost was positively correlated with advantageous inequality aversion and was negatively correlated with advantageous inequality aversion. This is inconsistent with the equality aversion model, which explains prosocial behaviors such as altruism (Fehr and Schmidt, 1999). The data from Spain also showed that individuals who were exposed to higher levels of inequality at the age of eight are more generous in adult life (Brañas-Garza et al., 2020). Based on the correlation of inequality aversion and social behavior, we investigated the effect of stimulation on inequality aversion. However, our results showed that right anodal/left cathodal stimulation and right cathodal anodal/left anodal stimulation did not change subjects’ inequality aversion. Moreover, we evaluated subjects’ inequality aversion immediately after the behavior experiments, and the Kruskal–Wallis test found no significant differences in inequality aversion across experiment 1 and experiment 2. Thus, DLPFC stimulation changed costly antisocial behavior but did not affect subjects’ inequality aversion, which is consistent with the findings presented in a previous study (Knoch et al., 2006). Ruff et al. (2013) also found that right lateral prefrontal cortex (rlPFC) stimulation did not affect one’s awareness of fairness norms. Our findings signify that although antisocial and antisocial behavior correlated with inequality aversion, DLPFC stimulation did not change inequality aversion.

Although our findings revealed that modulating DLPFC excitability altered participants’ costly antisocial behavior under the condition of inequality, the limitation of this study is that the neural circuitry underlying the decision-making process cannot be demonstrated by a single experiment. In addition, the inequality aversion task performed last and after tDCS. Further studies may take into consideration the inequality aversion task first. Furthermore, the involvement of other prefrontal areas, such as the ventromedial and anterior prefrontal cortex, is not discussed. Therefore, future studies may focus on the discussion of other brain regions and the neural circuit of the DLPFC. Moreover, the associations between other brain regions and costly and cost-free social behavior could also be further researched. The current study adopted bilateral stimulation; unilateral stimulation should be used as well in future studies. Furthermore, future studies should adopt neuroimaging measures and TMS to research neural changes associated with neuroimaging measures.

## Conclusion

Understanding the neurological mechanisms and brain regions associated with antisocial and prosocial behavior and the development of new interventions are important for both reducing violence and inequality. The current study revealed an asymmetric effect of bilateral stimulation over the DLPFC on costly antisocial behavior, while such an effect of antisocial behavior without cost and prosocial behavior with and without cost were not observed. Moreover, costly antisocial behavior exhibited by men receiving right anodal/left cathodal stimulation increased and decreased after receiving right cathodal anodal/left anodal stimulation compared with sham stimulation. However, subjects’ inequality aversion was not influenced by tDCS. We further found that costly antisocial behavior was positively correlated with disadvantageous inequality aversion and antisocial behavior without cost was positively correlated with disadvantageous inequality. In addition, costly prosocial behavior was positively correlated with advantageous inequality aversion. Prosocial behavior without cost was positively correlated with advantageous inequality aversion and was negatively correlated with advantageous inequality aversion.

## Data Availability Statement

The raw data supporting the conclusions of this article will be made available by the authors, without undue reservation.

## Ethics Statement

The studies involving human participants were reviewed and approved by the ethics committee of Zhejiang University of Finance and Economics. The patients/participants provided their written informed consent to participate in this study.

## Author Contributions

WZ, HY, YL, and JL designed and performed the experiments. WZ and JL analyzed the data and wrote the manuscript. WZ drew figures. All authors contributed to the article and approved the submitted version.

### Conflict of Interest

The authors declare that the research was conducted in the absence of any commercial or financial relationships that could be construed as a potential conflict of interest.
